# Manganese Deposition Encephalopathy Associated With Prolonged Total Parenteral Nutrition: A Case Report

**DOI:** 10.7759/cureus.117250

**Published:** 2026-09-30

**Authors:** Bruno Zanardo Faggion, Laura A Vicenzi Souza, Giancarlo Beluzzo da Mota de Lima Sinke, Rafaela Collaço de Oliveira Valente

**Affiliations:** 1 General Surgery, Hospital Universitário Polydoro Ernani de São Thiago, Universidade Federal de Santa Catarina (UFSC), Florianópolis, BRA; 2 Critical Care Medicine, Hospital Universitário Polydoro Ernani de São Thiago, Universidade Federal de Santa Catarina (UFSC), Florianópolis, BRA

**Keywords:** critical illness patients, extrahepatic cholestasis, manganese encephalopathy, manganese toxicity, parkinsonism, severe acute pancreatitis, total parenteral nutrition (tpn)

## Abstract

Manganese encephalopathy is a rare neurological disorder most commonly associated with chronic occupational exposure or impaired hepatobiliary elimination. We report a case of suspected manganese deposition encephalopathy associated with prolonged total parenteral nutrition in a critically ill patient with severe abdominal disease and cholestatic dysfunction. A 50-year-old man was admitted with severe abdominal pain, vomiting, tachycardia, and jaundice. Computed tomography demonstrated acute pancreatitis with pancreatic necrosis, choledocholithiasis, cholelithiasis, and superior mesenteric artery thrombosis. His clinical course was complicated by abdominal sepsis, anasarca, anemia, spontaneous hematomas, and the need for prolonged intensive care and total parenteral nutrition. On hospital day 41, he developed rigidity and involuntary movements involving the tongue and upper limbs, prompting neurological assessment. Serum manganese was 3.1 µg/L (reference value: 2 µg/L), and brain magnetic resonance imaging demonstrated T1 hyperintensity in the globus pallidus, findings compatible with manganese encephalopathy. Treatment with levodopa/benserazide produced a limited response, with persistent severe parkinsonism and inability to ambulate despite gradual improvement in tetraparesis associated with concurrent critical illness neuropathy. The patient was discharged on hospital day 129. This case highlights the importance of considering manganese toxicity in critically ill patients receiving prolonged parenteral nutrition, particularly in the setting of impaired biliary elimination, and supports early neurological surveillance and monitoring of trace elements.

## Introduction

Manganese is an essential trace element involved in several physiological processes, including enzymatic reactions, antioxidant defense, and cellular metabolism. Although manganese is required in small amounts, excessive systemic exposure or impaired elimination can result in neurological toxicity. Manganese encephalopathy is an uncommon neurological condition classically associated with chronic occupational exposure and advanced liver disease, in which impaired biliary excretion contributes to manganese accumulation and deposition within the central nervous system [1,2].

Patients receiving prolonged total parenteral nutrition (TPN) represent another clinically relevant population because manganese may be administered as part of trace-element supplementation. The risk of accumulation may be increased when biliary excretion is impaired [3]. We report a case of suspected manganese deposition encephalopathy in a critically ill patient who received prolonged TPN in the setting of severe abdominal disease, cholestatic dysfunction, and systemic inflammation.

This case was previously presented as a poster at the 36th Brazilian Congress of Surgery (CBC), held in Rio de Janeiro, Brazil, from August 7 to 10, 2025.

## Case presentation

A 50-year-old man presented to the emergency department of Hospital Universitário Polydoro Ernani de São Thiago, Universidade Federal de Santa Catarina, on day 1, with severe abdominal pain, vomiting, tachycardia, and jaundice. Computed tomography (CT) demonstrated acute pancreatitis, pancreatic necrosis, a 0.5 cm distal common bile duct choledocholithiasis, cholelithiasis, and superior mesenteric artery thrombosis. Laboratory testing revealed total bilirubin of 11.7 mg/dL, with a predominance of direct bilirubin (8.9 mg/dL), lipase of 394 U/L, amylase of 539 U/L, C-reactive protein of 182 mg/L, and leukocytosis (13.560 cells/mm^3^) with 12% band forms.

Urgent endoscopic retrograde cholangiopancreatography (ERCP) was indicated, in addition to antibiotic therapy (meropenem 1g 8/8h), nil per os (NPO), and anticoagulation. On day 4, ERCP demonstrated marked gastric stasis and severe duodenal edema, preventing visualization of the papilla. The patient was subsequently transferred to the intensive care unit. At that time, he had abdominal distension, diffuse abdominal pain, jaundice, and absent bowel sounds. A new CT scan was performed on day 3, showing no evidence of choledocholithiasis.

During the intensive care stay, the patient developed abdominal sepsis, anasarca, anemia, and spontaneous hematomas, requiring endotracheal intubation. TPN was initiated on hospital day 3. TPN was administered with daily trace-element supplementation at a dose of 2 mL/day. Each 2mL dose of the trace-element formulation contained 0.8 mg of manganese, corresponding to a daily parenteral manganese exposure of 0.8 mg. Throughout this period, the patient remained NPO, with no oral dietary intake. Enteral nutrition was intermittently introduced and advanced as clinically tolerated; however, it was repeatedly reduced or temporarily discontinued because of nausea, poor gastrointestinal tolerance, and difficulty in re-establishing enteral feeding in the setting of his ongoing critical illness. Consequently, enteral nutritional intake remained inconsistent, with alternating periods of enteral feeding and interruption, while TPN continued to provide nutritional support throughout this period. On hospital day 20, he underwent pancreatic necrosectomy. His subsequent clinical course was complicated by abdominal wound dehiscence with evisceration, requiring surgical re-exploration on day 27. Further surgical re-explorations were required on hospital days 32 and 35 because of recurrent intra-abdominal collections. He subsequently required additional interventions, including image-guided percutaneous drainage of persistent intra-abdominal collections.

On hospital day 41, the patient developed rigidity and involuntary movements, prompting the first neurological assessment. The movements initially involved the tongue and right upper limb and subsequently involved both upper limbs. At the time of examination, the patient was sedated in the intensive care unit, with a Richmond Agitation-Sedation Scale score of -3, limiting the assessment of mental status and cognition. The neurological examination documented clonic tongue movements, upper-limb tremor, and bilateral, symmetric upper-limb rigidity, most evident during extension. The pupils were miotic and reactive to light, and oculomotor and oculocephalic reflexes were present. Deep tendon reflexes were graded 2, with an expanded patellar reflexogenic area bilaterally. Hoffmann signs were present bilaterally, and plantar responses were indifferent.

The contemporaneous assessment did not document orientation, attention, memory, other cognitive domains, speech, a complete cranial nerve examination, numerical muscle strength grading, sensation, coordination, or gait and postural stability. Bradykinesia, hypomimia, and hypophonia were not documented at this initial assessment, although they were described subsequently. The upper-limb tremor was not characterized as occurring at rest, with posture, or during action. Dystonia and myoclonus were not explicitly documented, and the recorded clonic tongue movements were not further characterized. These undocumented examination components and movement phenomena were not retrospectively reconstructed, and lack of documentation was not interpreted as evidence of their absence.

The neurology team considered manganese accumulation associated with parenteral nutrition, metabolic disturbances, and adverse medication effects, and recommended brain MRI, medication review, and repeat electroencephalography. On hospital day 42, electroencephalography performed in the setting of reduced consciousness showed moderate background disorganization and frontal intermittent rhythmic delta activity (FIRDA). These findings were interpreted as indicating a nonspecific diffuse encephalopathy, potentially associated with medication-related, toxic-metabolic, or structural etiologies. Reactivity to stimulation was preserved, and no findings compatible with status epilepticus were identified. On hospital day 43, serum manganese was 3.1 µg/L, above the laboratory upper reference limit of 2.0 µg/L.

A subsequent medical report described brain MRI performed on hospital day 110 as showing intrinsic T1-weighted hyperintensity in the globus pallidus, interpreted in the clinical context as compatible with manganese deposition. Together with the neurological manifestations, elevated serum manganese concentration, and prolonged parenteral manganese exposure, the reported MRI findings supported the diagnostic hypothesis of manganese-associated encephalopathy. Representative images are presented in Figure 1.

**Figure 1:**
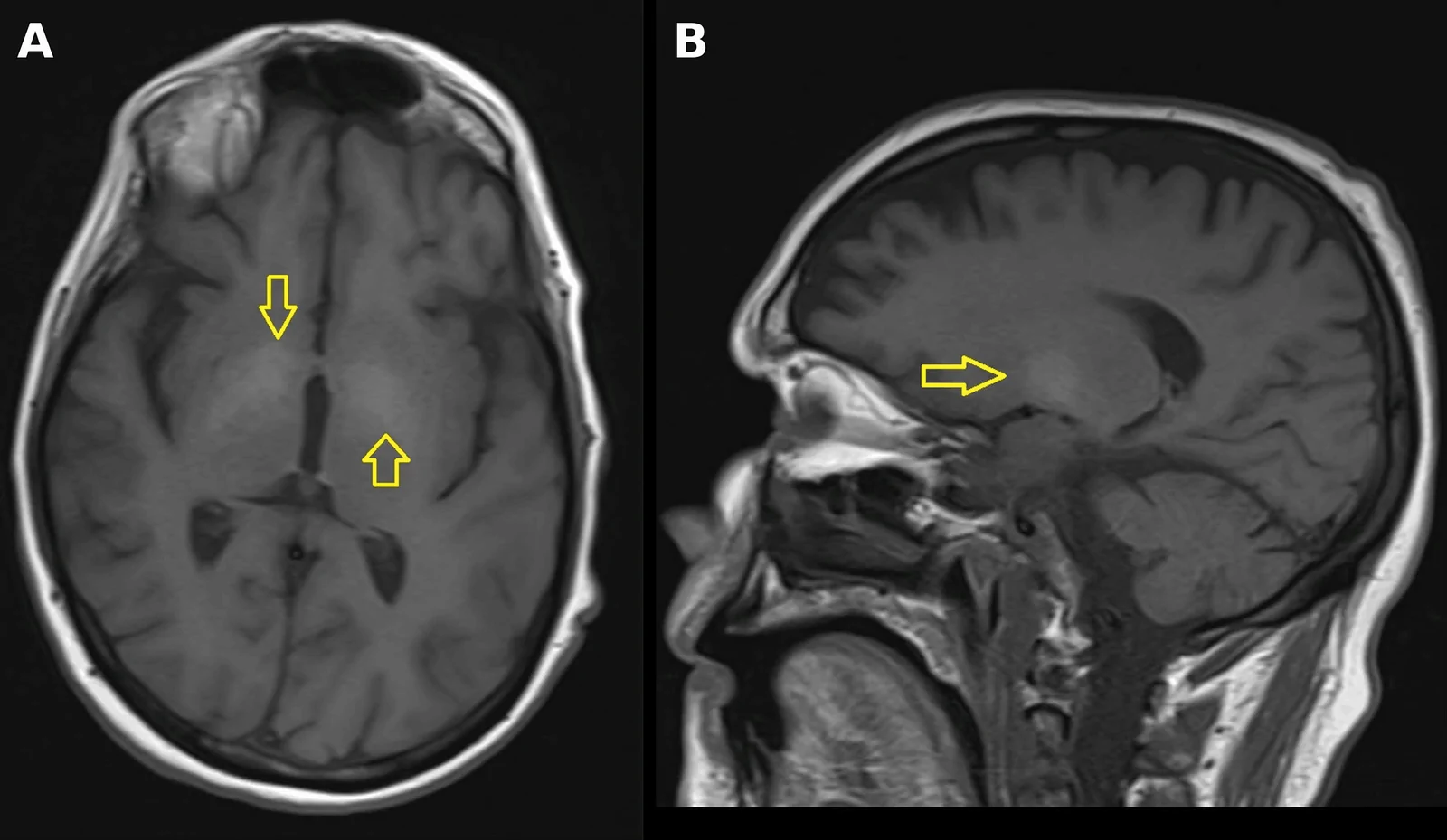
Image of T1-weighted hyperintensity in the globus pallidus. (A) Axial T1-weighted image demonstrating bilateral globus pallidus hyperintensity (yellow arrows). (B) Parasagittal T1-weighted image showing corresponding globus pallidus hyperintensity (yellow arrow).

On hospital day 110, TPN and trace-element supplementation were discontinued, enteral nutrition was increased, and levodopa/benserazide was initiated using 200 mg/50 mg tablets at a dose of one-half tablet three times daily. The dose was increased to one tablet three times daily on hospital day 115.

By hospital day 125, the documented neurological phenotype included cogwheel rigidity involving the upper and lower limbs, hypomimia, hypophonia, and severe, bilateral, symmetric generalized bradykinesia. The response to levodopa/benserazide was described as limited despite the regimen of 200 mg/50 mg three times daily. The Parkinsonian syndrome prevented ambulation, and the patient required a caregiver available 24 hours a day to assist with basic activities of daily living. Concurrent critical illness neuropathy with tetraparesis was also documented. The tetraparesis had gradually improved, although bilateral Achilles tendon shortening and marked limitation of active and passive ankle dorsiflexion persisted. No standardized parkinsonism rating scale or quantitative serial motor assessment was documented in the available records. No repeat manganese measurement or follow-up brain MRI was performed; therefore, biochemical normalization and radiological resolution could not be assessed. The patient was discharged on hospital day 129, with follow-up planned at the institutional movement disorders clinic.

## Discussion

Manganese encephalopathy is an uncommon toxic-metabolic neurological disorder characterized by manganese accumulation within the central nervous system. The condition has been described most extensively in individuals with chronic occupational exposure and in patients with impaired hepatic function, particularly when biliary excretion is compromised [1,2].

The present case is notable because the neurological syndrome developed during a prolonged critical illness requiring TPN. TPN can provide manganese as part of trace-element supplementation, and prolonged administration may contribute to manganese accumulation when the capacity for biliary elimination is reduced [3-5]. In this patient, the presence of jaundice and predominantly direct hyperbilirubinemia, together with severe abdominal disease and prolonged TPN, provided a clinically plausible setting for impaired manganese clearance. In this case, TPN was administered with a daily trace-element supplementation at a dose of 2 mL/day, providing 0.8 mg (800 μg) of manganese daily. This corresponded to approximately 14.5 times the currently recommended parenteral manganese intake of 55 μg/day for adults [6].

The neurological phenotype included cogwheel rigidity, severe bilateral symmetric bradykinesia, hypomimia, and hypophonia, supporting a parkinsonian syndrome, which is a recognized manifestation of manganese neurotoxicity [1,2]. Muscle weakness and impaired ambulation occurred concurrently but were also influenced by critical illness neuropathy and restricted ankle mobility. The biochemical and radiological findings further supported the diagnostic hypothesis. Serum manganese was elevated relative to the reported reference value, although blood manganese concentrations alone may have limited sensitivity and specificity for clinically apparent neurotoxicity [3]. Brain MRI demonstrated T1-weighted hyperintensity involving the globus pallidus, findings characteristic of manganese deposition in the basal ganglia [2,4]. The intensity of these abnormalities may correlate with manganese exposure and can decrease following reduction or withdrawal of manganese administration [7].

The bilateral pallidal T1-weighted hyperintensity was anatomically consistent with involvement of basal ganglia motor circuits and clinically concordant with the subsequently documented symmetric bradykinesia, cogwheel rigidity, hypomimia, and hypophonia. A similar combination of pallidal T1 hyperintensity and parkinsonian manifestations has been reported in patients receiving prolonged TPN [8]. In this patient, the concurrence of parenteral manganese exposure, an elevated serum manganese concentration, and compatible clinical and imaging findings supported manganese-associated encephalopathy. However, this clinicoradiological concordance did not establish manganese as the sole cause of neurological dysfunction. Concurrent critical illness neuropathy and restricted ankle mobility also contributed to motor disability, limiting attribution of the inability to ambulate exclusively to parkinsonism.

It is important to recognize that T1-weighted hyperintensity of the globus pallidus is not specific to clinically manifest manganese neurotoxicity and may also be observed in patients with chronic liver disease or impaired hepatobiliary function, reflecting altered manganese metabolism and clearance [9]. Acquired hepatocerebral degeneration (AHD) represents an important differential diagnosis, particularly in patients with advanced chronic liver disease or portosystemic shunting, as it may present with parkinsonian manifestations and similar bilateral T1-weighted hyperintensity of the globus pallidus associated with cerebral manganese accumulation [10]. However, AHD was considered less consistent with the present case, given the absence of known advanced chronic liver disease or portosystemic shunting and the clear temporal and clinical context of prolonged TPN containing manganese as part of trace-element supplementation, combined with cholestatic dysfunction that may have further impaired manganese elimination. Therefore, the overall clinical, biochemical, and radiological findings favored manganese accumulation associated with prolonged TPN in the setting of impaired hepatobiliary clearance.

Medication-induced parkinsonism was also considered, as the patient's usual medications included quetiapine and adverse medication effects were explicitly included in the initial neurological differential diagnosis. His usual medications, including quetiapine, were discontinued during hospitalization. However, the timing of withdrawal relative to symptom onset and any subsequent exposure to other dopamine receptor-blocking agents were not established in the available information. A medication-related contribution therefore could not be definitively excluded.

In this patient, severe abdominal sepsis and prolonged systemic inflammation occurred concurrently with TPN containing manganese supplementation and cholestatic dysfunction. Prolonged parenteral manganese exposure in the setting of impaired hepatobiliary elimination provided a plausible context for manganese accumulation [3]. However, sepsis-associated encephalopathy, sedative effects, and other metabolic disturbances were competing or potentially concurrent explanations for the early reduction in consciousness. Sedation at the initial neurological examination limited clinical assessment. EEG on hospital day 42 demonstrated background disorganization and FIRDA, with preserved reactivity and no findings compatible with status epilepticus. EEG abnormalities have been documented in septic ICU patients [11]; in this case, these findings indicated nonspecific cerebral dysfunction rather than a manganese-specific abnormality.

Although renal dysfunction occurred during hospitalization, the available data were insufficient to determine its temporal contribution to the neurological syndrome. No additional brain lesions were reported on MRI, but this did not exclude functional toxic-metabolic causes. The combination of documented parenteral manganese exposure, elevated serum manganese, persistent parkinsonian manifestations, and compatible pallidal T1 hyperintensity supported manganese-associated encephalopathy. Nevertheless, impaired manganese clearance due to cholestasis remained a proposed mechanism rather than a directly measured finding, and manganese could not be established as the sole cause of neurological impairment. No repeat manganese measurement or follow-up MRI was performed, precluding assessment of biochemical normalization or radiological resolution.

Recognition of manganese toxicity is clinically relevant because neurological manifestations may emerge insidiously in patients who are unable to communicate symptoms because of prolonged hospitalization, sedation, mechanical ventilation, or severe systemic illness. Monitoring of trace elements and early neurological assessment may therefore be particularly important in patients receiving prolonged TPN, especially when biliary or hepatic dysfunction is present [4,6,12].

Nagatomo et al. reported clinical and radiological improvement in two patients with TPN-associated manganese intoxication following discontinuation of manganese supplementation and treatment with levodopa [13]. In the present patient, however, the response to levodopa/benserazide was limited, with severe parkinsonism and inability to ambulate persisting on hospital day 125. The gradual improvement in tetraparesis attributed to concurrent critical illness neuropathy should be distinguished from the persistent parkinsonian manifestations. The absence of a specific chelating agent approved by the Brazilian Health Regulatory Agency (ANVISA), as noted in the original report, further emphasizes the importance of prevention, recognition, and management of modifiable risk factors.

## Conclusions

Manganese deposition encephalopathy should be considered in critically ill patients who develop new parkinsonian or motor manifestations during prolonged TPN, particularly when impaired biliary elimination is present. The combination of compatible neurological findings, elevated serum manganese, and characteristic basal ganglia abnormalities on T1-weighted MRI can support the diagnosis.

Early recognition may allow clinicians to reassess trace-element exposure and intensify neurological surveillance before more advanced neurological impairment develops. This case underscores the need for greater awareness of manganese toxicity as a potentially preventable complication of prolonged parenteral nutrition in critically ill patients.

## References

[1] Baj J, Flieger W, Barbachowska A (2023). Consequences of disturbing manganese homeostasis. Int J Mol Sci.

[2] Chen P, Chakraborty S, Mukhopadhyay S, Lee E, Paoliello MM, Bowman AB, Aschner M (2015). Manganese homeostasis in the nervous system. J Neurochem.

[3] Santos D, Batoreu C, Mateus L, Marreilha Dos Santos AP, Aschner M (2014). Manganese in human parenteral nutrition: considerations for toxicity and biomonitoring. Neurotoxicology.

[4] Livingstone C (2018). Manganese provision in parenteral nutrition: an update. Nutr Clin Pract.

[5] Martins AC Jr, Ruella Oliveira S, Barbosa F Jr (2021). Evaluating the risk of manganese-induced neurotoxicity of parenteral nutrition: review of the current literature. Expert Opin Drug Metab Toxicol.

[6] Berger MM, Shenkin A, Schweinlin A (2022). ESPEN micronutrient guideline. Clin Nutr.

[7] Takagi Y, Okada A, Sando K (2002). Evaluation of indexes of in vivo manganese status and the optimal intravenous dose for adult patients undergoing home parenteral nutrition. Am J Clin Nutr.

[8] Khan A, Hingre J, Dhamoon AS (2020). Manganese neurotoxicity as a complication of chronic total parenteral nutrition. Case Rep Neurol Med.

[9] Lim CG, Hahm MH, Lee HJ (2023). Hepatic encephalopathy on magnetic resonance imaging and its uncertain differential diagnoses: a narrative review. J Yeungnam Med Sci.

[10] Fernández-Rodriguez R, Contreras A, De Villoria JG, Grandas F (2010). Acquired hepatocerebral degeneration: clinical characteristics and MRI findings. Eur J Neurol.

[11] Azabou E, Magalhaes E, Braconnier A (2015). Early standard electroencephalogram abnormalities predict mortality in septic intensive care unit patients. PLoS One.

[12] Fell JM, Reynolds AP, Meadows N (1996). Manganese toxicity in children receiving long-term parenteral nutrition. Lancet.

[13] Nagatomo S, Umehara F, Hanada K, Nobuhara Y, Takenaga S, Arimura K, Osame M (1999). Manganese intoxication during total parenteral nutrition: report of two cases and review of the literature. J Neurol Sci.

